# A new nematode of the family Capillariidae identified in *Cairina moschata* (Linnaeus) on Marajó Island in the Brazilian Amazon

**DOI:** 10.1590/S1984-29612023047

**Published:** 2023-08-11

**Authors:** Elaine Lopes de Carvalho, Ricardo Luis Sousa Santana, José Ledamir Sindeaux, Michele Velasco Oliveira da Silva, Elane Guerreiro Giese

**Affiliations:** 1 Laboratório de Histologia e Embriologia Animal, Instituto da Saúde e Produção Animal, Universidade Federal Rural da Amazônia - UFRA, Belém, PA, Brasil; 2 Programa de Pós-graduação em Saúde e Produção Animal na Amazônia, Instituto da Saúde e Produção Animal, Universidade Federal Rural da Amazônia - UFRA, Belém, PA, Brasil; 3 Universidade Federal Rural da Amazônia - UFRA, Belém, PA, Brasil

**Keywords:** *Capillaria cairina* n. sp., Anseriformes, Muscovy duck, Brazil, *Capillaria cairina* n. sp., Anseriformes, Pato doméstico, Brasil

## Abstract

Capillaria Zeder, 1800, parasitizes the organs and tissues of several hosts, including the domestic duck *Cairina moschata* (Linnaeus). This article describes a new species of *Capillaria* in domestic ducks identified based on morphological studies and molecular analyses of the ribosomal RNA gene. Thirty-eight specimens of *C. moschata* from the municipality of Soure, Marajó Island, Pará, Brazil. The organs of the birds' digestive tract were analyzed under a stereomicroscope to confirm the parasitic infection, after which the collected nematodes were identified by light microscopy, scanning electron microscopy, and molecular analysis. Capillariids parasitized the intestine and cecum of the examined birds. These parasites had three bacillary bands and a pair of elongated precloacal papillae on the tail. Phylogenetic analysis indicated that the new species formed a sister clade with *Capillaria spinulosa* (Linstow, 1890), as described in Indonesia and Japan. Based on morphological distinctions and molecular data*, Capillaria cairina* n. sp. can be considered a new parasite species of *C. moschata* in the Brazilian Amazon.

## Resumo

*Capillaria* Zeder, 1800 parasitam órgãos e tecidos de diversos hospedeiros, incluindo o pato doméstico *Cairina moschata* (Linnaeus). Este artigo descreve uma nova espécie de *Capillaria* em patos domésticos, identificada com base em estudos morfológicos e análises moleculares do gene do RNA ribossomal. Trinta e oito espécimes de *C*. *moschata* oriundo dos no município de Soure, Ilha de Marajó, Pará, Brasil. Os órgãos do aparelho digestivo das aves foram analisados em estereomicroscópio para confirmar a infecção parasitária. Após, os nematódeos coletados foram identificados por microscopia de luz, microscopia eletrônica de varredura e análises moleculares. Os capilarídeos parasitaram intestino e ceco das aves examinadas. Esses parasitas apresentaram três bandas bacilares e um par de papilas pré-cloacais alongadas na cauda. A análise filogenética indicou que a nova espécie formou um clado irmão com *Capillaria spinulosa* (Linstow, 1890), conforme descrito na Indonésia e no Japão. Com base na distinção morfológica e dados moleculares, *Capillaria cairina* n. sp. pode ser considerada uma nova espécie de parasita de *C*. *moschata* na Amazonia brasileira.

## Introduction

Understanding the diversity and biology of nematodes of the family Capillariidae Railliet, 1915 remains scarce. Approximately 390 species have been identified in various organs and tissues in many hosts, such as fish, amphibians, reptiles, birds, and mammals, including humans (Vicente et al., 1995; Moravec, 2001a; Hodda, 2011). However, capillariids are often not identified in faunal surveys and have been reported only as *Capillaria* (*sensu latu*) sp. or Capillariidae gen. sp. (Moravec & Beveridge, 2017; Moravec & Justine, 2020).

The taxonomic classification and identification of capillariid nematodes are difficult as they are small, fragile parasites that require intense manipulation for observation (Moravec, 1982; Anderson, 2000; Tamaru et al., 2015). In addition, they exhibit a wide range of ecological adaptation mechanisms, parasitizing vertebrates belonging to the main classes in both terrestrial and aquatic conditions (fresh and marine water). Although many forms have adapted to life in the tissues of various organs (Moravec, 2001b), most capillariids are digestive tract parasites. Deng et al. (2021) demonstrated that using molecular markers to study phylogenetic and systematic relationships within the family Capillariidae is helpful in investigating controversial taxonomic problems in the identification of genera or species.

Muscovy ducks, *Cairina moschata* (Linnaeus), are well adapted to different climatic conditions and have adjusted to breeding in captivity (Béjcek & Stastný, 2002). However, they are also found in the wild, in areas with adequate water and space (Geromel, 2012). Subsistence farming is common in Brazil, and the trade of live birds, eggs, and meat occurs mainly among small producers, commercial houses, and open markets (Almeida et al., 2016). On Marajó Island, birds are raised extensively, making these animals generalists in their diet; thus, they can act as paratenic hosts by ingesting infected fish viscera or becoming infected after filtering water containing microcrustaceans parasitized by nematodes or eggs (Carvalho et al., 2020). On Marajó Island, Muscovy ducks are commonly used by the local population for food and commercial purposes (Carvalho et al., 2019, 2021).

In Brazil, four capillariid species have been identified among the parasitic fauna of *C. moschata* as follows: *Capillaria phasianina* Kotlán, 1914; *Capillaria* sp. Pinto and Almeida, 1935, and *Eucoleus cairinae* (Freitas & Almeida, 1935) Lopez & Neyra, 1947, in Rio de Janeiro; and *Eucoleus contortus* Creplin, 1989, in Pará (Vicente et al., 1995; Mattos et al., 2008; Carvalho et al., 2019). This study describes a new nematode species of the genus *Capillaria* Zeder, 1800, that was found to infect Muscovy ducks in the Brazilian Amazon based on morphological data and phylogenetic analyses.

## Material and Methods

### Sample collection

From 2018 to 2020, 38 *C*. *moschata* (25 females and 13 males) specimens were acquired from rural properties located in the municipality of Soure (00° 43′ 00″ S; 48° 31′ 24″ W), Marajó Island, Pará State. Only digestive tract organs were transported to the laboratory at the Universidade Federal Rural da Amazônia, Campus Belém, Pará.

### Parasitological examination

Each organ was isolated in a petri dish containing 0.9% NaCl saline in the laboratory and analyzed under a stereomicroscope (Leica ES2). Collected nematodes were washed in 0.9% saline, fixed in an A.F.A. solution (93 parts 70% ethyl alcohol, five parts formaldehyde, and two parts glacial acetic acid), and stored in 70% alcohol. In total, 197 adult nematodes were collected (114 females and 83 males), of which 118 were from the ceca, and 79 were from the large intestine. For light microscopy, nematodes were cleared in 0.5% lactophenol amine solution, observed under a Leica DM2500 microscope with a drawing tube, imaged under a Leica DM2500 microscope with a Leica DFC310 FX camera system using Leica Application Suite Software V4.4, and stored in glycerin alcohol (50% of 70% ethyl alcohol and 50% glycerin). For the morphometric analysis, 20 males, 20 females, and 50 eggs were used. Measurements are given in micrometers unless otherwise indicated and are represented as mean values, followed by the minimum and maximum values in parentheses.

### Scanning electron microscopy (SEM)

Forty-five nematodes (22 females and 23 males) were fixed in 3% glutaraldehyde, washed in 0.2 M phosphate buffer solution, post-fixed in 1% osmium tetroxide for 2 h, dehydrated in progressive alcohol for 1 h each (50%, 70%, 80%, 90%, and 100%), dried at the critical point of CO_2_, metalized with gold-palladium, and observed using a Vega 3 (TESCAN, Brno, Czech Republic) scanning electron microscope, according to Carvalho et al. (2022).

### Molecular analysis

For molecular and phylogenetic analyses, 20 nematodes (10 females and 10 males) were used. Helminths were isolated from the ceca and fixed in absolute alcohol. Total DNA was extracted using the Purelink^®^ Genomic DNA Mini Kit (Invitrogen^®^; ThermoFisher, CA, USA), following the manufacturer’s instructions. The small subunit ribosomal RNA gene (SSU rDNA) sequence was amplified using primers 18S-E/18S-A27 and 18S-8/Cestode-6 (Olson & Caira, 1999). The final volume for the polymerase chain reaction (PCR) was 25 μL, containing 1 ng of DNA template, 20 mM Tris pH 8.4, 50 mM KCl, 2 mM dNTP (Invitrogen^®^), 1 mM Mg_2_Cl, 0.5 pmol of each primer, and 0.2 U of Taq DNA polymerase (Invitrogen^®^). The amplification profile for the polymerization of any molecules that might have dissociated from the polymerase prior to complete fragment synthesis consisted of 5 min of initial denaturation at 95 °C, followed by 35 cycles of 1 min at 94 °C, 1 min at 60 °C, and 1 min at 72 °C, with a final extension of 7 min at 72 °C.

The amplicons were subjected to 1.5% agarose gel electrophoresis and purification using ExoSAP-ITTM (GE Healthcare, UK). Quantification was performed using Nanodrop (ThermoFisher, CA, USA). The samples were sequenced using an Applied Biosystems^™^ 3500 Genetic Analyzer (ThermoFisher, CA, USA), which generated approximately 700 nucleotides in each sequence. The primers used for PCR amplification and sequencing were as follows: 18S-E forward (5′-CCG AAT TCG ACA ACC TGG TTG ATC CTG CCA GT-3′)/18S-A27 reverse (3′-CCA TAC AAA CGT CCC CGC CTG -5′) and 18S-8 forward (5′-GCA GCC GCG GTA ATT CCA GC-3’)/18S-Cestode 6 reverse (3’-ACG GAA ACC TTG TTA CGA CT-5′).

The nucleotide sequences obtained from the samples were edited and aligned using BioEdit software (Hall et al., 2011) after a comparison with other sequences available in GenBank (BLAST search). The SSU rDNA sequence was aligned with sequences of 21 capillariids available in GenBank. In addition, the database includes sequences from *Haemonchus placei* (Place, 1893) and *Haemonchus contortus* (Rudolphi, 1803), which formed the outgroup for phylogenetic analyses. The consensus nucleotide sequences reported in this study are available in GenBank under accession number OP720889.

Bayesian inference (BI) was performed using a Markov Chain Monte Carlo (MCMC) phylogenetic tree, implemented in MrBayes 3.1.2 (Ronquist & Huelsenbeck, 2003). This analysis was based on two parallel runs of four simultaneous MCMC searches of five million generations each. One tree was sampled every 250 generations after discarding the first 1000 trees as burn-in. The remaining trees were analyzed using MrBayes to estimate the posterior probability of each node in the phylogenetic reconstruction. As indicated by jModelTest 2.1.9 (Darriba et al., 2012), the BI analysis assumed a TIM3ef + I + G nucleotide substitution model, with estimated base frequencies (A = 0.2571, C = 0.2091, G = 0.2751, and T = 0.2587), the replacement model (A-C = 0.6282, A-G = 2.3543, A-T = 1.0599, C-G = 0.6321, C-T = 3.8687, G-T = 1.0000), and local variables after a gamma distribution (G = 0.3820), and 88 models in the 100% confidence interval. In addition, genetic distances were determined for the SSU rDNA sequences of the capillariid species using PAUP 4.0 (Swofford, 1998). Specimens of capillariids were deposited in the Coleção Helmintológica do Instituto Oswaldo Cruz (CHIOC), Manguinhos, Rio de Janeiro, Brazil, as follows: holotype (CHIOC 39393 a), allotype (CHIOC 39393 b), and paratypesfour males (CHIOC 39393 c-f) and four females (CHIOC 39393 g-j).

## Results

### Description

Trichinellida Hall, 1916

Capillariidae Railliet, 1915

*Capillaria* Zeder, 1800

**Figure 1:**
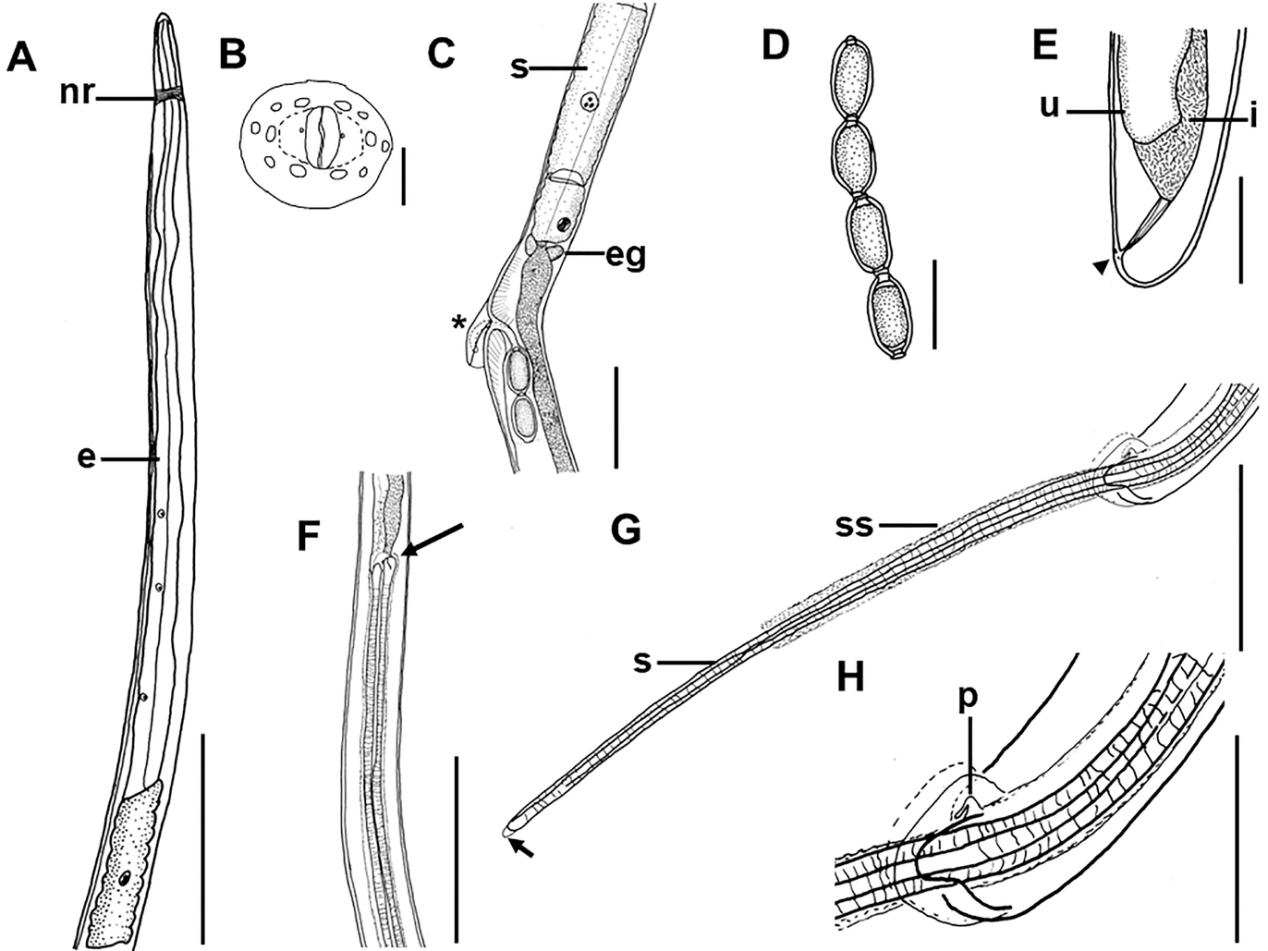
*Capillaria cairina* n. sp. (A) Anterior extremity, muscular esophagus (e), and nerve ring (nr). Scale bar = 10 µm; (B) Detail of the male’s anterior extremity, prominent lips surrounded by 12 papillae, apical view (reconstructed from an SEM micrograph). Scale bar = 2 µm; (C) End of stichocytes (s), esophageal glands (eg), and pre-equatorial region where the vulva and vulvar appendix are located (*). Scale bar = 10 µm; (D) Eggs. Scale bar = 40 µm; (E) Female posterior extremity, lateral view, anal opening (arrowhead); the uterus (u) and intestine (i) are shown. Scale bar = 50 µm; (F) Region where the base of the spicule is observed (arrow). Scale bar = 10 µm; (G) Posterior extremity of male, lateral view, spicule (s), spinous spicular sheath (ss), and rounded tip of the spicule (arrow). Scale bar = 10 µm. H. Posterior extremity of male showing papilla (p). Scale bar = 5 µm.

**Figure 2:**
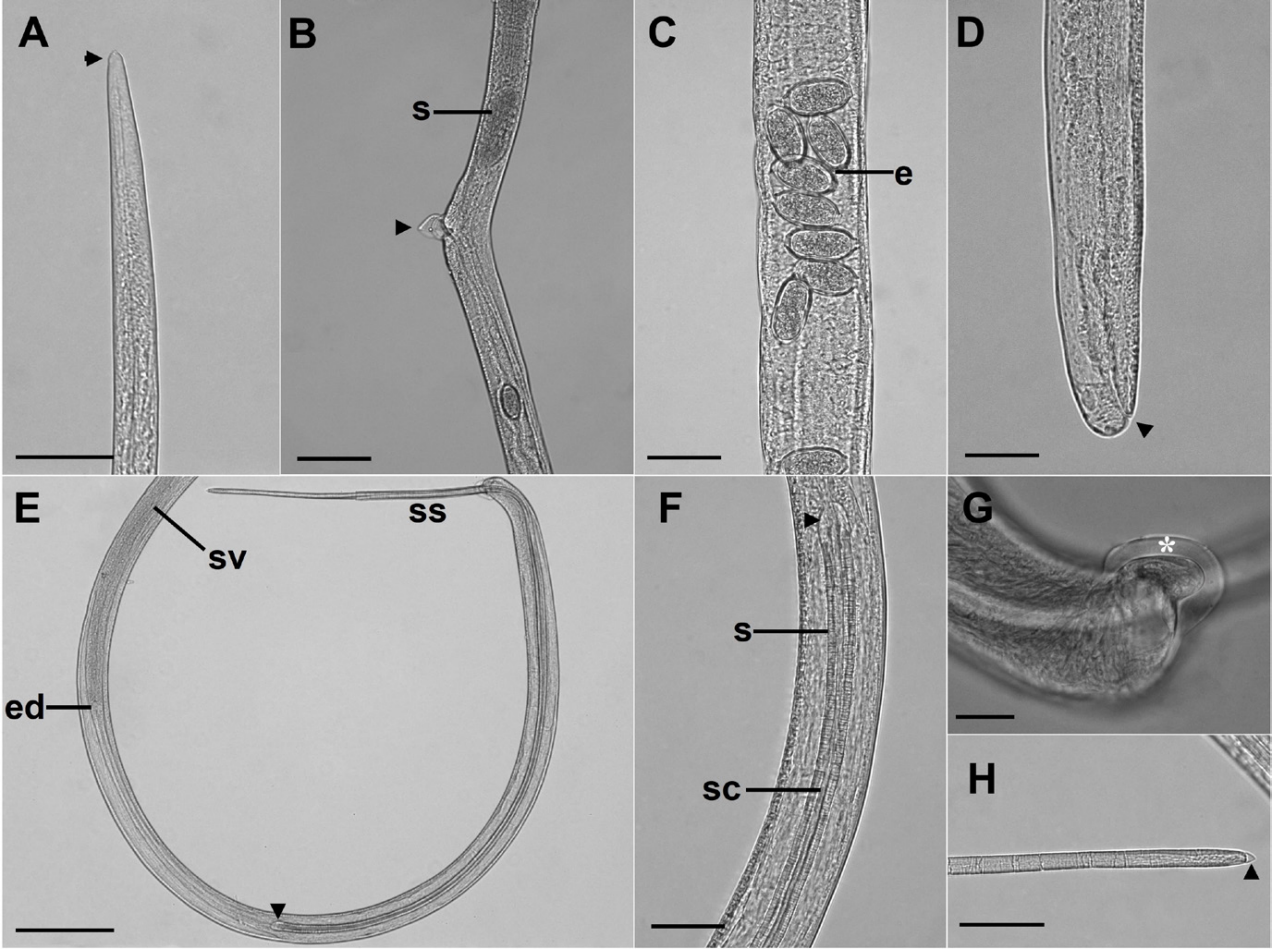
Microscopic views of *Capillaria cairina* n. sp. (A) Female anterior end (arrowhead). Scale bar = 50 µm; (B) Pre-equatorial region, lateral view, where the stichocytes (s) end and the vulva with its vulvar appendix (arrowhead). Scale bar = 100 µm; (C) Uterus with eggs (e) with clearly visible polar plugs. Scale bar = 50 µm; (D) Posterior extremity, lateral view; the anal opening is observed (arrowhead). Scale bar = 50 µm; (E) Posterior extremity of the male, lateral view; the base of the spicule (arrowhead) and extruded spicular sheath (ss) can be observed. Scale bar = 200 µm; (F) Base of the spicule (arrowhead). Scale bar = 50 µm; (G) Posterior extremity of the male, lateral view, lobe with a delicate membrane (*). Scale bar = 20 µm; (H) Distal end of the spicule with a rounded tip and the presence of a thin membrane at its apex (arrowhead). Scale bar = 50 µm. s = spicule; ss = spicular sheath; sc = spicular canal; ed = ejaculatory duct; sv = seminal vesicle.

**Figure 3:**
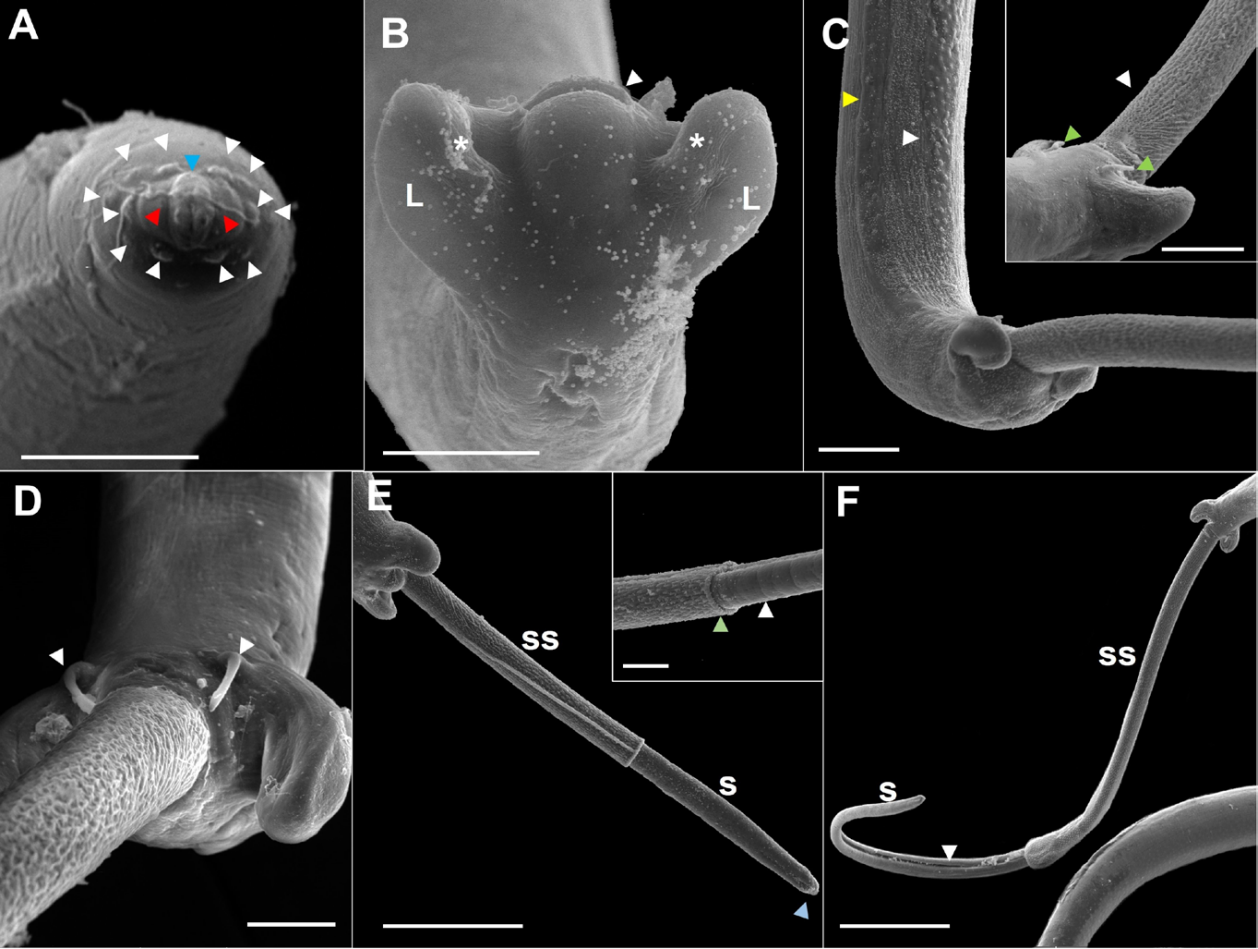
Scanning electron microscopy of male *Capillaria cairina* n. sp. (A) Detail of the male’s anterior extremity, prominent lips (blue arrowhead) surrounded by 12 papillae (white arrow), apical view, and pair of small lateral amphids (red arrowhead). Scale bar = 5 µm; (B) Posterior extremity, ventral view, cloacal opening (arrowhead), membrane (*) and the caudal lobes (L). Scale bar = 20 µm; (C) Posterior extremity, lateral and ventral views of the tail with an extruded spicular sheath; lateral (yellow arrowhead) and ventral (white arrowhead) bacillary bands can be observed. Scale bar = 20 µm. In the insert, note the digitiform pre-cloacal papillae (green arrowhead) and the base of the spineless spicular sheath (white arrowhead). Scale bar = 20 µm; (D) Note the digitiform pre-cloacal papillae (arrowhead). Scale bar = 10 µm; (E) Posterior end with partially extruded spinous spicular sheath (ss), rough spicule (s), and membrane of the distal end of the spicule (blue arrow). Scale bar = 50 µm. Insert showing the distal end of the spicular sheath with spines (green arrowhead) and rough spicule (arrowhead). Scale bar = 10 µm; (F) Posterior extremity with spicule (s) and spicular sheath (ss) with fully extruded spines; the spicular canal is observed (arrowhead). Scale bar = 100 µm.

**Figure 4:**
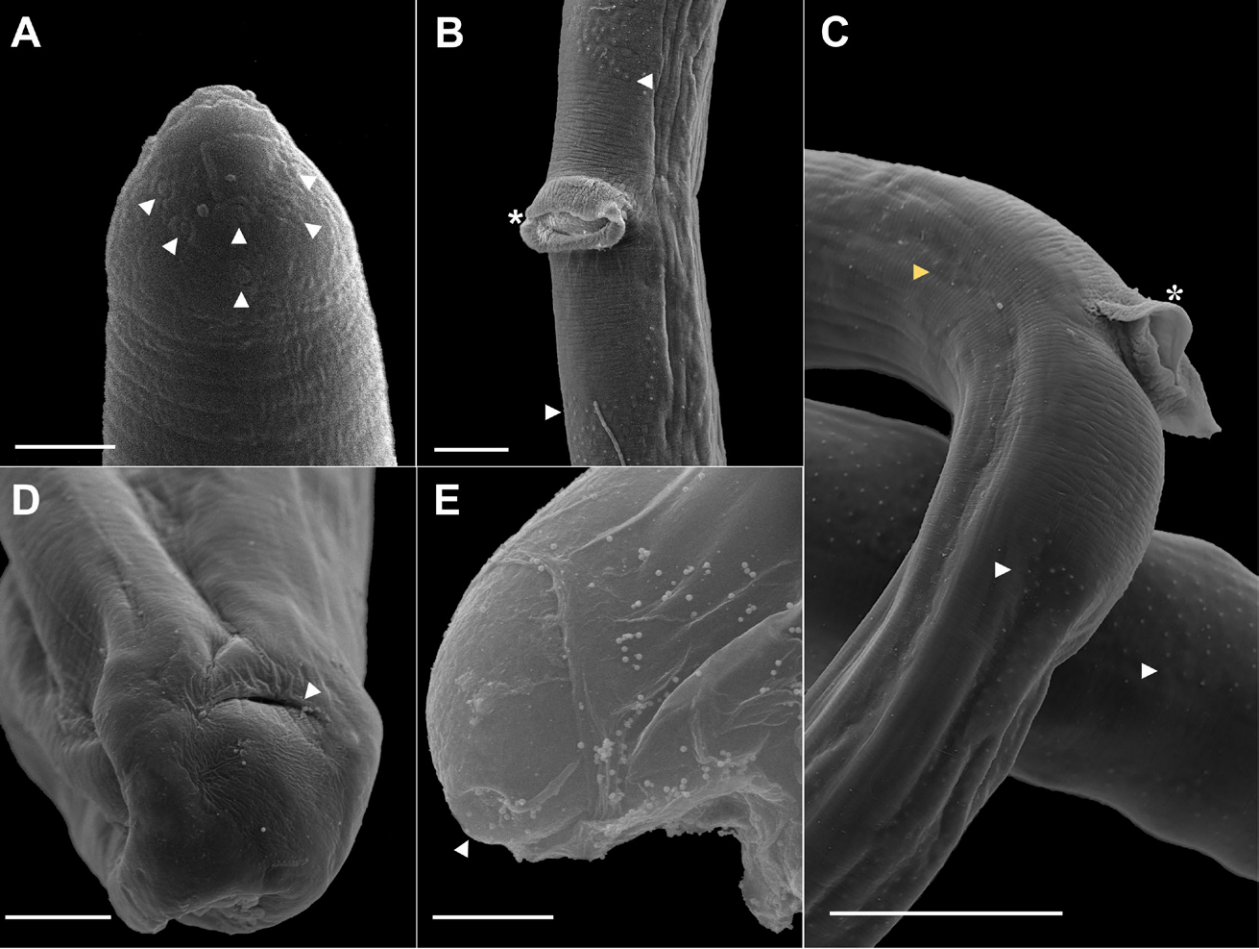
Scanning electron microscopy of female *Capillaria cairina* n. sp. (A) Anterior end, large papillae (white arrowhead). Scale bar =2 µm; (B) The vulva is observed in the pre-equatorial region, with the presence of a vulvar appendix (*) and a rectangular area of interruption of the ventral bacillary bands (arrowhead). Scale bar = 20 µm; (C) Lateral (yellow arrowhead) and ventral (white arrowhead) views of the bacillary band and observed vulvar appendix (*). Scale bar = 50 µm; (D) Posterior extremity, lateral and ventral views of the tail; the anal opening is observed (arrowhead). Scale bar = 10 µm; (E) Observation of the egg with a polar plug (arrowhead) still in the vulvar appendix. Scale bar = 10 µm.

*Capillaria cairina* n. sp.

(Based on light microscopy and SEM, Figures 1-4)

The nematodes were small and filiform, with delicate cuticles that were transversely striated. They exhibited a simple, dorsoventrally oriented oral opening. Their mouth was surrounded by 12 cephalic papillae arranged in two circles, each consisting of six papillae and a pair of small lateral amphids, with a stylet absent. They contained a short, muscular esophagus with a stichosome. The nerve ring surrounded the muscular esophagus in its initial portion. The stichosome consisted of a single row of approximately 40 elongated stichocytes with transverse rings that were difficult to visualize and large stichocytes nuclei with several nucleoli. Two glandular cells were observed at the esophageal-intestinal junction. Lateral and ventral bacillary bands extended for almost the entire body length in both sexes (Figures 1A-B, G).

Male measurements (based on 20 specimens with an extruded spicular sheath, holotype measurements in brackets) were as follows: body length, 14.7 (11.8-15.8) mm [15.3 mm]; maximum width at the esophageal-intestinal junction, 43 (36-51) [45]; muscular esophagus measuring 335 (256-366) [338] × 14 (13-15) [15] wide; bacillary bands lateral and ventral to the body; total esophagus length, 5.7 (5.0-6.2) mm [6.2 mm], representing 39 (35-41) % [41%] of the body length; stichosome length, 5.4 (4.7-5.9) mm [5.9 mm]; the number of stichocytes, 41 (37-41) [39]; 13 (7-17) [7] transverse rings. The nerve ring was situated 69 (41-70) [70] from the anterior extremity. They exhibited a single, heavily sclerotized spicule measuring 1.7 (1.5-1.7) mm [1.6 mm] × 22 (16-23) [23] wide, representing 12 (10-11) % [10%] of the body length, with a spinous spicular sheath measuring 280 (123-433) [286] × 18 (13-16) [16]. The sheath was not spiny at the base. A well-developed spicular canal was observed. The proximal end of the spicule was expanded, and the distal end was rounded, with rough transverse grooves observed on the spicule surface. The caudal end was rounded and bifurcated in ventral and dorsal views, without a pseudobursa, supporting two large, round ventrolateral lobes containing a pair of sessile ventrolateral pre-cloacal papillae and a terminal cloacal opening (Figures 1F-G, 2E-H, 3A-F).

Female measurements (based on 20 gravid specimens, allotype measurements in brackets) were as follows: body length, 23.1 (16.7-28.6) mm [24.6 mm]; maximum width at the esophageal-intestinal junction, 45 (38-53) [45]; bacillary bands lateral and ventral to the body; total length of the esophagus, 6.7 (5.4-8.0) mm [7.9 mm], representing 29 (26-33) % [32%] of the body length; length of the muscular esophagus, 380 (335-445) [401] × 15 (13-20) [13] wide; stichosome length, 6.3 (5.1-7.6) mm [7.4 mm]; the number of stichocytes, 40 (37-42) [40], with 14 (11-23) [12] transverse rings. The nerve ring was situated 72 (46-95) [76] from the anterior extremity. The pre-equatorial vulva was located 7.7 (5.5-12.0) mm [7.9 mm] from the anterior extremity, with vulvar lips exhibiting appendages. The vagina was short and muscular. Eggs were barrel-shaped and arranged in a single row near the vulvar passageway, measuring 42 (35-48) [42] × 20 (18-22) [20] with projecting polar plugs. Females exhibited rounded caudal ends with a subterminal anus (Figures 1B-D, 2B-D, 4A-E).

### Molecular and phylogenetic analyses

**Figure 5:**
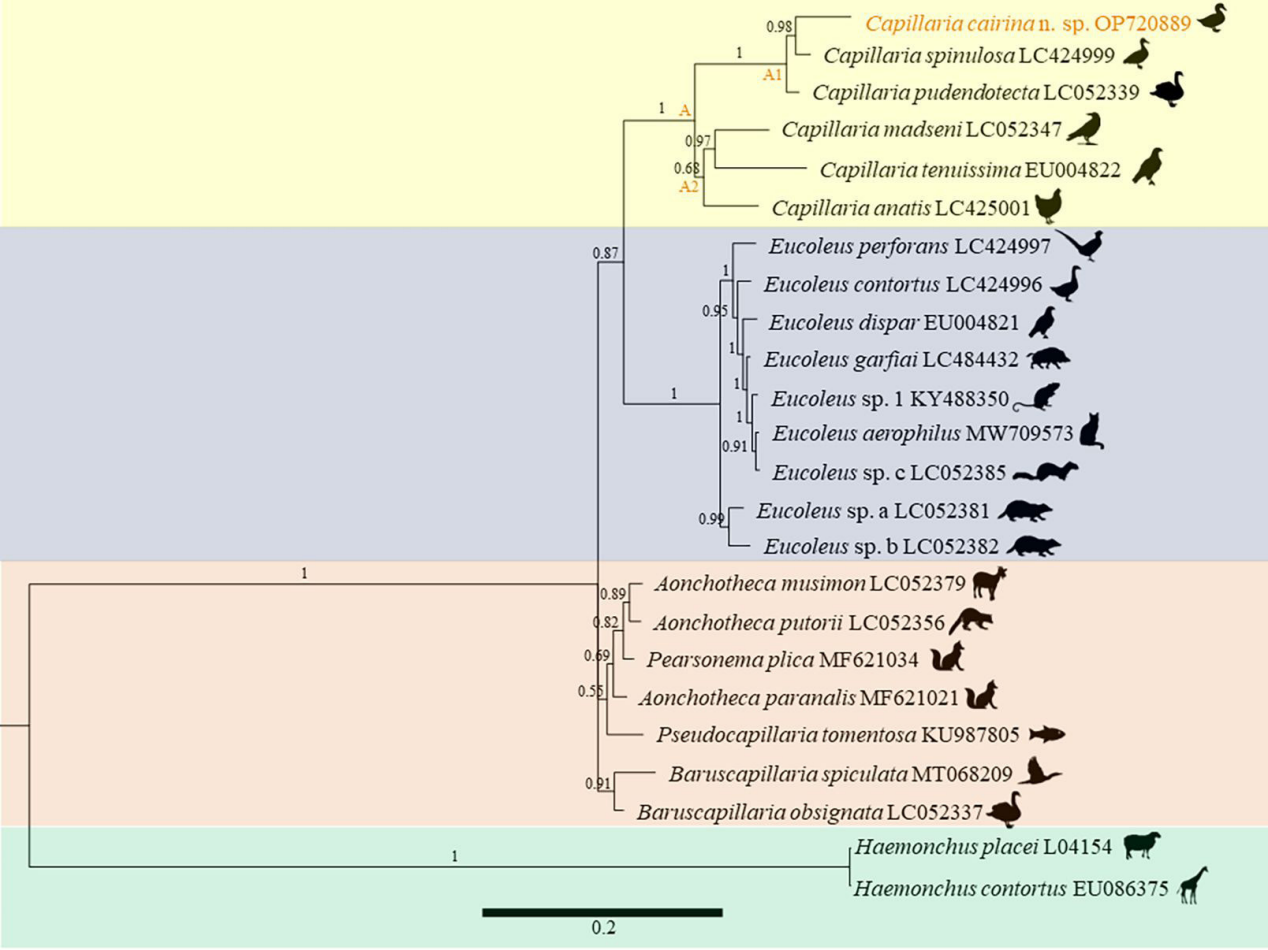
Bayesian phylogenetic tree based on SSU rDNA sequence obtained from *Capillaria cairina* n. sp., compared to that of other capillariids. Node numbers represent probability values calculated from BI/bootstrap ML values (> 50%). The scale bar indicates the number of mutations per site. Data are displayed with the species names, followed by the images of the hosts.

The partial rDNA sequence obtained for *Capillaria cairina* n. sp. was 1750 bp in length and is available in GenBank (OP720889). Thirty-one taxa were used for the comparison, and the outgroups were *H. contortus* and *H. placei*. Among these, four large, well-supported clades were formed as follows: A (*Capillaria*), B (*Eucoleus* Dujardin, 1845), C (predominantly *Aonchotheca* López-Neyra, 1947), and D (*Baruscapillaria*Moravec, 1982). A BLAST search revealed that the nucleotide sequences with the highest similarity were those of *Capillaria pudendotecta* Lubimova, 1947 (accession numbers LC052338 and LC052339), observed in swans in Japan, with 95.45% and 95.53% similarity, respectively, and of *Capillaria spinulosa* (Linstow, 1890) (accession numbers LC424999 and LC425000), in domestic birds from Indonesia and Japan, with 95.32% similarity. Clade A formed two subclades, A1 and A2, in which parasites belonging to the genus *Capillaria* were observed in the following hosts: Anseriformes (Anatidae), Galliformes (Phasianidae), Passeriformes (Corvidae), and Accipitriformes (Accipitridae), with infection sites in the ceca and small intestine (Figure 5).

Remarks: The analysis of clade A and subclades A1 and A2 was performed, as the parasites were of the same genus as those in our study and differed due to the morphological characteristics observed in Table 1, with SEM and molecular biology being fundamental for the identification of *Capillaria cairina* n. sp.

**Table 1:** Data on the distance *p* calculated for the capillariid species.

	1	2	3	4	5	6	7	8	9	10	11	12	13	14	15	16	17	18	19	20	21	22
Present study	-																					
*Capillaria madseni*	0.171	-																				
*Capillaria pudendotecta*	0.064	0.153	-																			
*Capillaria anatis*	0.163	0.099	0.135	-																		
*Capillaria tenuissima*	0.218	0.136	0.194	0.153	-																	
*Capillaria spinulosa*	0.060	0.150	0.029	0.151	0.201	-																
*Eucoleus* sp. a	0.264	0.212	0.231	0.195	0.251	0.243	-															
*Eucoleus* sp. b	0.271	0.215	0.246	0.207	0.247	0.25	0.028	-														
*Eucoleus* sp. 1	0.207	0.145	0.165	0.144	0.217	0.191	0.027	0.030	-													
*Eucoleus* sp. c	0.285	0.232	0.250	0.215	0.274	0.261	0.039	0.043	0.004	-												
*Eucoleus contortus*	0.263	0.210	0.232	0.197	0.248	0.241	0.039	0.041	0.012	0.021	-											
*Eucoleus dispar*	0.270	0.232	0.261	0.217	0.270	0.271	0.043	0.053	0.008	0.020	0.023	-										
*Eucoleus aerophilus*	0.288	0.230	0.253	0.213	0.271	0.262	0.040	0.045	0.003	0.003	0.020	0.021	-									
*Eucoleus garfiai*	0.275	0.206	0.241	0.192	0.248	0.252	0.035	0.040	0.004	0.010	0.017	0.015	0.008	-								
*Eucoleus perforans*	0.276	0.222	0.247	0.214	0.268	0.259	0.046	0.048	0.021	0.029	0.030	0.034	0.031	0.027	-							
*Aonchotheca paranalis*	0.236	0.156	0.193	0.158	0.214	0.203	0.142	0.135	0.120	0.161	0.143	0.158	0.159	0.148	0.160	-						
*Aonchotheca musimon*	0.239	0.161	0.188	0.153	0.213	0.203	0.129	0.130	0.109	0.148	0.136	0.147	0.148	0.146	0.139	0.033	-					
*Aonchotheca putorii*	0.237	0.153	0.191	0.148	0.211	0.205	0.127	0.129	0.113	0.152	0.130	0.149	0.151	0.146	0.139	0.031	0.019	-				
*Pearsonema plica*	0.233	0.154	0.194	0.151	0.202	0.201	0.136	0.128	0.113	0.152	0.137	0.145	0.152	0.141	0.146	0.024	0.021	0.022	-			
*Pseudocapillaria tomentosa*	0.222	0.147	0.190	0.165	0.223	0.199	0.152	0.145	0.126	0.163	0.151	0.160	0.164	0.145	0.165	0.045	0.051	0.051	0.046	-		
*Baruscapillaria spiculata*	0.251	0.152	0.205	0.159	0.217	0.217	0.164	0.164	0.118	0.186	0.166	0.170	0.184	0.160	0.177	0.070	0.073	0.074	0.067	0.074	-	
*Baruscapillaria obsignata*	0.205	0.123	0.157	0.121	0.181	0.170	0.114	0.114	0.102	0.128	0.117	0.131	0.128	0.140	0.125	0.039	0.039	0.038	0.038	0.044	0.036	-

*Capillaria cairina* n. sp., observed in subclade A1 in this study, formed a sister clade with *C*. *spinulosa* with a genetic distance of 6%, which can be justified as these parasites infect hosts of the same Anseriformes order and Anatidae family. *Capillaria cairina* n. sp. has morphological characteristics that distinguish it from *C*. *spinulosa* (Table 1), and it is the only family in this clade to occur in the ceca and large intestine of Muscovy ducks in Brazil. In this subclade, *C*. *pudendotecta* had a genetic distance of 6.4% to the results of our study. However, the only similarity was observed in Anseriformes and Anatidae birds.

Subclade A2 contained *Capillaria anatis* (Schrank, 1790), *Capillaria madseni* Wakelin, Schmidt & Kuntz, 1970, and *Capillaria tenuissima* (Rudolphi, 1803) (with genetic distances of 16%, 17.1%, and 21,8%, respectively), which are parasites of Galliformes observed in the ceca and small intestine. In contrast, Accipitriformes and Passeriformes birds from Japan and Germany were infected only in the small intestine, thus differing from in our study.

### Taxonomic summary

**Host type**: *Cairina moschata* (Anseriformes: Anatidae).

**Host site**: large intestine and ceca.

**Location**: Municipality of Soure (00° 43′ 00″ S; 48° 31′ 24″ W), State of Pará, Brazilian Amazon.

**Parasite data**: prevalence, 47.4%; total worm recovery, 197; intensity, 2-38 (10.9 on average); abundance, 5.18.

**Etymology**: The specific name *cairina* (genitive) is related to the generic name of the host bird (*Cairina*).

## Discussion

Baruš et al. (1978) recorded three species of *Capillaria* parasitizing piscivorous birds from Europe and Asia based on the site of infection and length of the male’s spicular sheath. Thus, the species *C. anatis*, *Capillaria spirale* (Molin, 1858) Travassos, 1915, and *Capillaria contorta* (Creplin, 1839) were identified parasitizing birds of the orders Podicipediformes, *Podiceps grisegena* (Boddaert); Anseriformes, *Mergus merganser* (Linnaeus) and *Mergusserrator* (Linnaeus); and Charadriiformes, *Larus argentatus* (Pontoppidan)*, Larusminutus* (Pallas), *Larusridibundus* (Linnaeus), *Hydroprogne caspia* (Pallas), *Thalasseus sandvicensis* (Latham), and *Sternula albifrons* (Pallas). Despite the wide distribution of these parasites in birds, few studies still identify these capillariids at the specific level (Dar et al., 2013; Stapf et al., 2013; Carvalho et al., 2019).

According to Moravec (1982) and Vicente et al. (1995), the genus *Capillaria* has the following diagnostic characteristics: the caudal lateral wings are absent in males; the posterior end of the male is rounded and equipped with two lateral lobes, located ventrolaterally or dorsolaterally. This genus lacks a membranous bursa, and sessile pre-cloacal papillae are often observed. These nematodes exhibit a sclerotized spicule and spiny spicular sheath, and they may or may not exhibit a vulvar appendix. They are intestinal parasites of fish, amphibians, reptiles, birds, and mammals, with thetype species being *C. anatis*. The parasite observed in *C*. *moschata* in this study exhibited characteristics of *Capillaria*. Baruš et al. (1981) identified *C*. *anatis* in *Anas acuta* (Linnaeus) and observed that the posterior end of the male forms a pseudobursa with a smooth cuticle and that the base is formed by two well-developed and rounded lateral processes, with a rounded cloacal opening located ventrally. The species identified in our study had a pair of sessile, longitudinally elongated pre-cloacal papillae, transverse cloacal openings, and lobes without pseudobursa; however, each had a well-marked membrane (Figure 2G).

By light microscopy, the parasite seemed to be similar to *C*. *anatis*, *C*. *spinulosa*, and *C*. *pudendotecta*. However, with SEM, our capillariid showed three bacillary bands (lateral and ventral), a delicate membrane in each lobe, and a pair of elongated pre-cloacal papillae, which differed from the descriptions of *C*. *anatis* reported by Baruš et al. (1978, 1981). They observed microscopically the posterior end of males, and they could observe a delicate membrane between ventro-lateral lobes, called “pseudobursa,” and a pair of short pre-cloacal papillae in males. The description differed from that of *C*. *spinulosa*, provided by Mettrick (1959) and Sakaguchi et al. (2020), who observed males with a caudal end showing two small, non-elongated papillae. Oyarzún-Ruiz et al. (2019) identified the presence of a spiny sheath with a distal expansion without a thorn in males of *C*. *pudendotecta*, which differed from the description of males in our study (Figure 4F) since the absence of spines was at the base of the sheath.

All female specimens in our study were morphologically similar to *C*. *pudendotecta* described by Oyarzún-Ruiz et al. (2019), with a small vulvar appendix. However, they could be easily distinguished from *C*. *anatis* and *C*. *spinulosa* described by Baruš et al. (1981), Tanveer et al. (2015), and Yevstafieva et al. (2020). They identified females without a vulvar appendix, and Mettrick (1959) and Sakaguchi et al. (2020) reported that *C*. *spinulosa* females had a vulva without a vulvar appendix. These differed from the females in our study but corroborated Moravec's (2001b) observations, who reported that capillariid females may or may not have a vulvar appendix. Thus, the organisms identified in this study differed from those already registered (Table 2).

**Table 2:** Comparison of the morphometric characteristics of *Capillaria cairina* n. sp. with other species of the genus *Capillaria* that present spicular sheath in Brazil found in the intestines and ceca of birds.

Morphometric characterization	*Capillaria cairina* n. sp.		*C. anatis*		*C. anatis*		*C. longicollis*		*C. nyrocinarum*		*C. spinulosa*		*C. tenuissima*	
Specimen sex	**Male**	**Female**	Male	Female	Male	Female	Male	Female	Male	Female	Male	Female	Male	Female
Host	***Cairina moschata***		*Anas querquedula, Anas crecca, Anas platyrhynchos, Fulica atra*		*Anser anser anser*		Pigeon, little bustard and several gallinaceous birds		Eider - ducks		Wigeon and common scoter		Owls	
Locality	**Soure, Pará, Brazil**		Zoological Museum of Copenhagen, Denmark		Hertfordshire		British Museum		Escotland		British Museum		Britain	
Total body (L) ^a^	**11.8**-**15.8**	**16.7-28.6**	7.0-12.2	8.4-14.9	6.2-10.7	7.1-12.9	8.4-14.7	8.9-17.6	9.7-11.6	7.6-13.4	7.8-8.5	9.8-13.1	8.7-11.3	18.2-23.3
Maximum body (W) ^b^	**36**-**51**	**38-53**	-	-	54	66	68	73	78	88	48	62	59	69
Nerve-ring (L) ^b, c^	**41**-**70**	**46-95**	-	-	-	-	-	-	-	-	-	-	-	-
Muscular esophagus (L) ^b^	**256-366**	**335-445**	-	-	-	-	-	-	-	-	-	-	-	-
Muscular esophagus (W) ^b^	**13-15**	**13-20**	-	-	-	-	-	-	-	-	-	-	-	-
Total esophagus (W) ^a, c^	**5.0-6.2**	**5.4-8.0**	-	-	-	-	-	-	-	-	-	-	-	-
Vulva (L) ^a^	**–**	**5.5-12.0^c^**	-	2.1-2.7^c^	-	0.036-0.042^d^	-	0.030-0.070^d^	-	-	-	0.066-0.072^d^	-	-
Vulvar appendage	**–**	**Present**	-	-	-	Absent	-	Present	-	Present	-	Absent	-	Absent
Egg (L × W) ^b^	**35-48 × 18-22**		49-65 × ?		-		49-58 × 22-24		59-62 × 30-32				60-64 × 30-34	
Spicule (L) ^a^	**1.5-1.7**	-	1.22-1.83	-	1.22-1.37	-	0.96-1.02	-	1.5-7.5	-	0.64-0.71	-	0.68-0.71	-
Spicule (W) ^b^	**16-23**	-	-	-	20-22	-	-	-	10-12	-	18	-	14-15	-
Spicular sheath spines	**Present**	-	-	-	Absent	-	Present	-	Present	-	Present	**-**	Present	-
Spicular sheath (L × W) ^b^	**123-433 × 13-16**		-	-	-	-	?0.010-0.012	-	0.025	-	**-**	-		-
# Specimen	**20**	**20**	28	11	-	-	-	-	20		3	7	-	-
Reference	**Present study**		Madsen (1945)		Mettrick (1959)		Mettrick (1959)		Mettrick (1959)		Mettrick (1959)		Mettrick (1959)	
Morphometric characterization	*Capillariacairina* n. sp.		*C. ovopunctata*		*C. collaris*		*C. phasianina*		*C. anatis*		*C. caudinflata*		*C. anatis*	
Specimen sex	**Male**	**Female**	Male	Female	Male	Female	Male	Female	Male	Female	Male	Female	Male	Female
Host	***Cairina moschata***		Blackbirds and starlings		*Odontophorus capueira capueira*		*Phasianus colchicus torquatus*		Domestic fowl		British Domestic fowls		*Anas platyrhynchos*	
Locality	**Soure, Pará, Brazil**		Hertfordshire		Brazil		Brazil		Great Britain		Inglaterra		UK	
Total body (L) ^a^	**11.8**-**15.8**	**16.7-28.6**	6.84-10.3	11.2-14.0	8.3	-	17.82-19.77	26.97-38.76	6.70-13.14	8.11-18.34	8.80-17.60	11.88-25.38	11.99-14.46	15.87-24.69
Maximum body (W) ^b^	**36**-**51**	**38-53**	52	62	66	-	66	70-83	35-58	44-60	33-51	38-62	-	-
Nerve-ring (L) ^b, c^	**41**-**70**	**46-95**	-	-	76	-	84-92	105-130	-	-	-	-	-	-
Muscular esophagus (L) ^b^	**256-366**	**335-445**	-	-	206	-	365-522	435-566	-	-	-	-	-	-
Muscular esophagus (W) ^b^	**13-15**	**13-20**	-	-	-	-	-	-	-	-	-	-	-	-
Total esophagus (W) ^a, c^	**5.0-6.2**	**5.4-8.0**	-	-	3.82	-	6.87-8.04	7.37-8.78	4.23-5.29	4.23-6.70	4.59-7.05	4.59-7.41	-	-
Vulva (L) ^a, c^	–	**5.5-12.0^c^**	**-**	0.050-0.057^d^	-	-	**-**	0.042-0.097^d^	-	**-**	**-**	-	**-**	**-**
Vulvar appendage	–	**Present**	**-**	Present	-	-	**-**	-	-	**-**	**-**	Present	**-**	**-**
Egg (L × W) ^b^	**35-48 × 18-22**		57-58 × 27-28		-		46-63 × 21-29		55-62 × 22-29		47-58 × 20-24		53-58 × 24-29	
Spicule (L) ^a^	**1.5-1.7**	-	0.91 - 0.94	-	0.96	-	1.74-2.41	-	1.06-1.86	-	0.71-1.25	-	1.40-1.97	-
Spicule (W) ^b^	**16-23**	-	3-8	-	17	-	17-29	-	14-22	-	3-5	-	-	-
Spicular sheath spines	**Present**	-	Present	**-**	Present	-	Present	**-**	Present	**-**	Present	**-**	Present	-
Spicular sheath (L × W) ^b^	**123-433 × 13-16**		-	**-**	17 × 21		? × 25-34		-		**-**		-	
# Specimen	**20**	**20**	-	-	1	-	5	5	100	100	100	100	20	20
Reference	**Present study**		Mettrick (1959)		Freitas et al. (1959)		Freitas et al. (1959)		Wakelin (1965)		Wakelin (1965)		Wakelin (1965)	
Morphometric characterization	*Capillariacairina* n. sp.		*C. anatis*		*C. anatis*		*C. anatis*		*C. anatis*		*C. anatis*		*C. anatis*	
Specimen sex	**Male**	**Female**	Male	Female	Male	Female	Male	Female	Male	Female	Male	Female	Male	Female
Host	***Cairina moschata***		*Perdix perdix*		*Anas acuta*		*Corvus monedula; C. splendens*		Ducks Anatinae		*Gallus gallus domesticus*		Chicken	
Locality	**Soure, Pará, Brazil**		Collection of the London		Delta, USSR		Kashmir, India		North-Western Poland		Kashmir valley		Kagoshima, Japan	
Total body (L) ^a^	**11.8**-**15.8**	**16.7-28.6**	-	19.32	6.70-13.14	8.11 - 18.34	8.20-13.5	9.42-16.50	7.71-10.55	6.83-14.04	10.5-12.9	14.3-18.5	6.42-9.97	7.25-16.58
Maximum body (W) ^b^	**36**-**51**	**38-53**	-	-	35-58	44 - 60	28-60	30-64	35-42	40-50	60-90	51-140	36-64	50-80
Nerve-ring (L) ^b, c^	**41**-**70**	**46-95**	-	-	-	-	-	-	-	-	-	-	-	-
Muscular esophagus (L) ^b^	**256-366**	**335-445**	-	-	-	-	-	-	-	-	-	-	-	-
Muscular esophagus (W) ^b^	**13-15**	**13-20**	-	-	-	-	-	-	-	-	-	-	-	-
Total esophagus (W) ^a, c^	**5.0-6.2**	**5.4-8.0**	-	-	4.23-5.29	4.23 - 6.70	4.46-5.85	4.10-6.05	-	-	4.23 - 6.25	4.12-6.0	3.44-5.06	3.00-6.56
Vulva (L) ^a, c^	**–**	**5.5-12.0^c^**	-	-	-	-	-	4.46-6.12^c^	-	-	**-**	4.34-4.56^c^	-	0.011 × 0.083^d^
Vulvar appendage	**-**	**Present**	-	-	-	-	-	-	-	-	**-**	-	-	-
Egg (L × W) ^b^	**35-48 × 18-22**		57 × 26		-		48-57 × 24-27		40-52 × 22-25		51-65 × 25-30		53-63 × 24-34	
Spicule (L) ^a^	**1.5-1.7**	-	1.52	-	1.06-1.86	-	0.48-0.65	-	0.63-0.92	-	1.0-1.2	-	0.73-1.21	-
Spicule (W) ^b^	**16-23**	-	-	-	-	-	13-24	-	-	-	14-25	-	-	-
Spicular sheath spines	**Present**	-	-	-	Present	-	Present	-	Present	-	Present	**-**	Present	**-**
Spicular sheath (L × W) ^b^	**123-433 × 13-16**		-	-	-	9 × 10	140-180×?		-	-	-		**-**	-
# Specimen	**20**	**20**	2	2	10	10	10	20	4	11	28		28	29
Reference	**Present study**		Wakelin (1967)		Baruš et al. (1981)		Dar et al. (2013)		Stapf et al. (2013)		Tanveer et al. (2015)		Tamaru et al. (2015)	
Morphometric characterization	*Capillaria cairina* n. sp.		*C. anatis*		*C. anatis*		*C. spinulosa*		*C. spinulosa*		*C. phasianina*		*C. amatis*	
Specimen sex	**Male**	**Female**	Male	Female	Male	Female	Male	Female	Male	Female	Male	Female	Male	Female
Host	***Cairina moschata***		Chicken		Chicken		*Anas platyrhynchos*		*Anser cygnoides*		*Phasianus colchicus versicolor*		*Anas platyrhynchos domesticus*	
									*domesticus*					
Locality	**Soure, Pará, Brazil**		Davao Oriental, Philippines		Indonesia		Surabaya, Indonesia		Surabaya, Indonesia		Kumamoto, Japan		Ukraine	
Total body (L) ^a^	**11.8**-**15.8**	**16.7-28.6**	7.69-14.06	12.61-20.83	10.95-13.97	13.56-14.23	15.00-16.71	24.4	12.34-14.58	10.06-23.61	19.29	19.77	7.20-9.80	9.70-12.50
Maximum body (W) ^b^	**36**-**51**	**38-53**	50-72	60-106	53-65	55-58	55-57	71	38-52	52-73	65	65	44.78-47.21	58.17-61.28
Nerve-ring (L) ^b, c^	**41**-**70**	**46-95**	-	-	-	-	-	-	-	-	-	-	–	–
Muscular esophagus (L) ^b^	**256-366**	**335-445**	-	-	-	-	-	-	-	-	-	-	–	–
Muscular esophagus (W) ^b^	**13-15**	**13-20**	-	-	-	-	-	-	-	-	-	-	–	–
Total esophagus (W) ^a, c^	**5.0-6.2**	**5.4-8.0**	3.50-6.69	4.97-6.94	4.76-5.77	5.24-5.48	5.88-6.74	6.65	5.27-5.84	5.18-7.89	5.48	5.44	–	–
Vulva (L) ^a,^^c^	-	**5.5-12.0^c^**	-	0.011-0.117^d^	-	0.023-0.068^c^	-	-	-	0.058-0.427^d^	-	-	–	4.45-6.15
Vulvar appendage	-	**Present**	-	-	-	-	-	-	-	-	-	-	–	Absent
Egg (L × W) b	**35-48 × 18-22**		49-66 × 26-37		54-59 × 24-29		45 × 24		40-50 × 21-24		41-49 × 24-25		56.29-61.25 × 29.45-35.41	
Spicule (L) ^a^	**1.5-1.7**	-	0.89-1.12	-	0.94-1.15	-	0.65-0.72	-	0.74-0.78	-	2.55	-	1.40-1.64	–
Spicule (W) b	**16-23**	-	-	-	-	-	-	-	-	-	-	-	10.20-11.62	–
Spicular sheath spines	**Present**	-	Present	-	-	-	Present	-	Present	-	Present	**-**	Absent	–
Spicular sheath (L × W) b	**123-433 × 13-16**		-	-	-	-	-	-	-	-	**-**	-	–	
# Specimen	**20**	**20**	21	18	3	2	3	1	3	3	1	1	15	15
Reference	**Present study**		Tamaru et al. (2015)		Sakaguchi et al. (2020)		Sakaguchi et al. (2020)		Sakaguchi et al. (2020)		Sakaguchi et al. (2020)		Yevstafieva et al. (2020)	

a Measurements in millimeters;

b Measurements in micrometers;

c Calculated from anterior extremity;

d Calculated from end esophagus.

Abbreviations: L = length. W = width.

Note: #: numbers.

Tamaru et al. (2015) and Sakaguchi et al. (2020) recorded *C*. *pudendotecta* and *C*. *spinulosa*, respectively, only in the ceca of *Anas platyrhynchos* var. *domesticus* (Linnaeus), *Anser cygnoides domesticus* (Linnaeus), and *Cygnus olor* (Gmelin). The morphometric comparisons with other capillariids that parasitize the large intestine and ceca of birds are described in Table 2. Yevstafieva et al. (2020) reported specific morphological characteristics and biometric parameters of male and female *С*. *anatis* observed in *A. platyrhynchos* raised on poultry farms in Ukraine. As reported by Moravec (1982, 2001b) and Melnychuk et al. (2020), males have characteristics such as a pseudobursa, spicules, and spicular sheath ornamentation, and in females, the morphology of the vulvar area and eggs in the uterus should be considered.

Cram (1936) and Moravec (2001b) considered the shape and arrangement of hypodermic cells (bacillary bands) as a complementary characteristic for the differentiation between genera and species of capillaries. In the capillariids identified in this study, three bacillary bands were observed in both males and females, which differed from those observed in other species. In addition, the male was reported to have a spicular sheath with sclerotized spines oriented toward the cloaca. The spines were sparse in the proximal part of the spicule sheath, whereas they were more densely distributed in the middle and distal parts.

The morphological details of capillariids, such as the anterior extremity, bacillary bands, and tail, are not visible under light microscopy (Moravec, 2001a; Moravec & Barton, 2018; Carvalho et al., 2019). In this study, we observed the conical shape of the cephalic region with large and flat papillae in both males and females using SEM. Females have a well-defined vulvar appendix and a pair of papillae close to the vulva. In addition, a large “rectangular” area without a bacillary band was observed around the vulva. The tail of the male exhibited a pair of thin and elongated papillae, a spicular sheath armed with small spines, and three bacillary bands, with the ventral bacillary band being wider in females. None of these aspects have been previously reported in capillariid species and are described for the first time in the genus *Capillaria* in our study (Figures 3C-D).

The descriptions of many capillariids and the establishment of unjustified new genera within the family Capillariidae have confused the taxonomy and systematics of this group of parasites (Moravec et al., 1981). Sakaguchi et al. (2020) constructed a phylogenetic tree based on the SSU rDNA nucleotide sequences of representative species of the Capillariidae family reported in birds and mammals. All species shown in the tree were grouped according to the genera described by Moravec (1982), confirming that the morphological classification in our study matches the species identified in the phylogenetic analysis of Sakaguchi et al. (2020). Phylogenetic analyses have become a complementary part of studies involving species identification, thus establishing current genetic relationships between capillariid species. This helps elucidate their complex taxonomy and phylogenetic arrangements to corroborate the existing taxonomic classification (Tamaru et al., 2015; Borba et al., 2019; Deng et al., 2021).

The pairwise genetic distance between our isolate and *C*. *spinulosa* was 6%, this distance is considered high because most distances between capillariid species belonging to the same genus are less than 1%, according to Tamaru et al. (2015), Sakaguchi et al. (2020) and Garbin et al. (2021). Sakaguchi et al. (2020) noted that integrative approaches are highly recommended for identifying capillariids. Thus, in the phylogenetic tree constructed in our study, the species were grouped according to the site of infection, order, and host family. These factors determine the degree of proximity between the *Capillaria* species analyzed in this study.

## Conclusion

Through morphological, morphometric, and phylogenetic analyses of the capillariid nematodes in our study, we identified a new species, *Capillaria cairina* n. sp., which parasitizes the large intestine and ceca of *C*. *moschata*.
